# Study of re-transplantation and prognosis in liver transplant center in Iran 

**Published:** 2021

**Authors:** Javad Salimi, Ali Jafarian, Nasir Fakhar, Alireza Ramandi, Mohamad Behzadi, Ali Moeni, Habib Dashti, Atabak Najafi, Mohammad Reza Shariat, Jalil Makarem, Abdolhamid Chavoshi Khamneh

**Affiliations:** *Liver Transplantation Centre, Imam Khomeini Hospital, Tehran University of Medical Siences, Tehran, Iran *

**Keywords:** Liver re-transplant, Prognosis, Survival

## Abstract

**Aim:**

This this study aimed to investigate the causes and prognoses of liver re-transplantation in patients referred to Imam Khomeini Hospital Liver Transplantation Center.

**Background:**

Organ shortage is a major problem in the world, a high demand for liver transplantation has exacerbated this problem. Thus, providing more information on the causes of liver re-transplantation, its prognosis, and other issues related to this procedure is of great importance.

**Methods:**

This study was conducted in 2018 as a historical cohort. In this study, the records of liver transplantation patients at Imam Khomeini Hospital Liver Transplantation Center between 2000 and 2016 were studied, and data was extracted from the records of patients undergoing liver transplantation. Patient data was entered into SPSS 20 software and analyzed.

**Results:**

In this study, 1030 patients with a mean age of 43.15 ± 14.57 years were studied. There were 426 women (41.4%) and 604 men (58.6%). The number of primary transplants was 966 with a mean age of 43.19 ± 14.72, and the number of re-transplants was 64 with a mean age of 42.56 ± 12.82. Significant differences were found between the two groups in terms of MELD and CHILD scores, cold ischemic time, total and direct bilirubin levels, liver function factors (ALT, AST, and alkaline phosphatase), hemoglobin, and WBC. There was no significant difference between the two groups in terms of age, sex, or platelets (> 0.05). The mortality rate was 241 (23.39%) in all patients and the mortality rate was 206 (21.32%) and in liver transplant patients was 35 (54.68%). The mortality rate in the transplant group was statistically higher (*p*<0.001). Secondary was primary non-functional graft (PNF) (37.5%) with 1-, 3, and 5-year survival rates of 82%, 81%, and 70% in primary group and 59%, 43% and 32% in re-transplantation, respectively. There was a significant difference in survival between the two groups (*p* <0.05). Hemoglobin and alkaline phosphatase were predictors of survival rates in transplant patients.

**Conclusion:**

The results of this study showed that the survival rate of re-transplant patients was significantly lower than that of primary transplant patients, and the mortality rate in re-transplant patients was significantly higher.

## Introduction

 Being the only treatment method for patients with advanced liver failure, liver transplantation was first performed on a human in 1963 by a surgical team led by Thomas Starzel in the United States (1). According to the literature, the failure rate of liver allograft is between 14% and 23% (2, 3). Although controversial, the only treatment for graft failure is liver re-transplant (Re-LT). However, there are more severe complications following Re-LT compared to first time liver transplant (LT-I). Moreover, the one-year survival rate of recipients of Re-LT patients is approximately 60%, compared to 80-90% after LT-I (4, 5). 

The necessity of Re-LT exacerbates the problem of organ shortage and high demand. Therefore, obtaining more information about the causes of Re-LT, its prognosis, and other issues related to this process is very important. Because of poorer outcomes, several studies have examined the risk factors for morbidities and mortality after Re-LT. Hong et al. identified factors such as age, MELD score, mechanical ventilation, serum albumin, age, blood intake, and number of days after the first transplant that Re-LT was required as risk factors that impair prognosis; those having no such factors had a 5-year survival rate of 79% (5). The issue of re-transplantation in other organs has also had poorer outcomes compared to the original transplant. Re-transplantation of the kidney and pancreas had significantly less survival than the primary transplant (6, 7). Recipients of heart and lung re-transplantation also had less overall survival than the primary transplant (8-10).

Previous studies have given several reasons for liver transplantation (11), and the outcome and prognosis for liver transplantation have varied depending on the reason for liver transplantation (12). Consequently, the aim of this study was to investigate the causes of liver transplantation and the survival rate and factors influencing the prognosis of liver transplantation. 

## Methods

This study was performed as a retrospective cohort using the electronic files of transplanted patients between 2000 and 2016. This study was carried out with the approval of the Ethics Committee of Tehran University of Medical Sciences (code of ethics: IR.TUMS.IKHC.REC.1397.194). Included in this study were all patients over 18 years of age who were transplanted at our center. Patients who had transplantation of other organs before, during, or after liver transplantation were excluded. The demographic data, laboratory data, primary and re-transplantation indications, MELD score, CHILD score, mean cold ischemic time (CIT), and mortality of each patient were recorded.

Data was analyzed using SPSS software. Qualitative analysis was reported in prevalence and percentage. Quantitative analysis was reported as mean ± standard deviation (SD). Comparisons between different groups in terms of classification variables were performed using the chi-square test. In case of correction, the Fisher Exact test was used. For data with normal distribution, comparison between variables with different groups was performed using the independent t-test. For non-parametric variables, comparisons between variables were performed using the Mann-Whitney test. Survival analysis of patients was performed by the Kaplan-Meire method. To determine the factors affecting re-transplant rejection, univariant linear regression was utilized. The level of significance was considered as less than 0.05.

**Table 1:** Demographic information

Variable		All patients (n=1030)	Primary LT(n=966)	Secondary LT(n=64)	P-value
Age (Year)		43.15±14.57	14.72 ± 43.19	12.82 ± 42.56	0.698
Gender	Female	(%41.4) 426	(%93.2)397	(%6.8)29	0.51
	Male	(%58.6)604	(%94.2)569	(%5.8)35	
MELD		6.00 ± 20.72	5.63 ± 20.51	10.73 ± 25.89	0.004
CHILD		2.05 ± 9.96	2.05 ± 9.98	1.65 ± 8.75	0.04
Cold ischemic time (min)		88.07 ± 300.64	87.41 ± 304.42	78.00 ± 243.10	<0.001
Lab	Total bilirubin	8.00 ± 6.81	7.60 ± 6.61	13.70 ± 11.28	0.03
	Direct bilirubin	5.25 ± 3.66	5.12 ± 3.55	7.13 ± 6.13	0.02
	AST	348.83 ± 130.24	261.86 ± 110.39	1055.01 ± 565.12	0.009
	ALT	336.27 ± 105.17	297.31 ± 89.95	741.59 ± 438.17	0.005
	ALKp	477.93 ± 454.39	381.64 ± 433.16	1359.70 ± 915.86	0.029
	WBC (×10^3^)	8.75 ± 6.65	8.81 ± 6.52	6.69 ± 9.59	0.03
	Hb	2.01 ± 11.39	1.99 ± 11.45	2.05 ± 10.22	<0.001
	Platelet (×10^3^)	85.43 ± 102.44	85.61 ± 102.02	81.87 ± 111.76	0.476
Mortality		241	(%21.32)206	(54.68 %)35	<0.001

## Results

Between 2000 and 2016, 1,030 patients were transplanted. Mean patient age was 43.15 ± 14.57 years. There were 426 women (41.4%) and 604 men (58.6%) investigated in this study. The number of primary transplant cases and re-transplant cases were 966 and 64, respectively. Patients undergoing primary and re-transplantation were similar in terms of age and gender. A comparison of patients who have undergone primary transplantation to patients with re-transplantation showed that the MELD score, total and direct bilirubin levels, liver function factors (ALT, AST and ALKP), hemoglobin, and WBC were significantly higher in re-transplant patients; however, CHILD score and cold ischemic time (CIT) were significantly lower in re-transplant patients, and platelet count was similar in both groups. The mortality rate was 241 patients (23.39%), of whom 206 expired patients were in the primary transplant group and 35 patients were in the re-transplant group. Statistically, the mortality rate was significantly higher in transplant patients (*p*<0.001) (Table 1).

**Figure 1:**
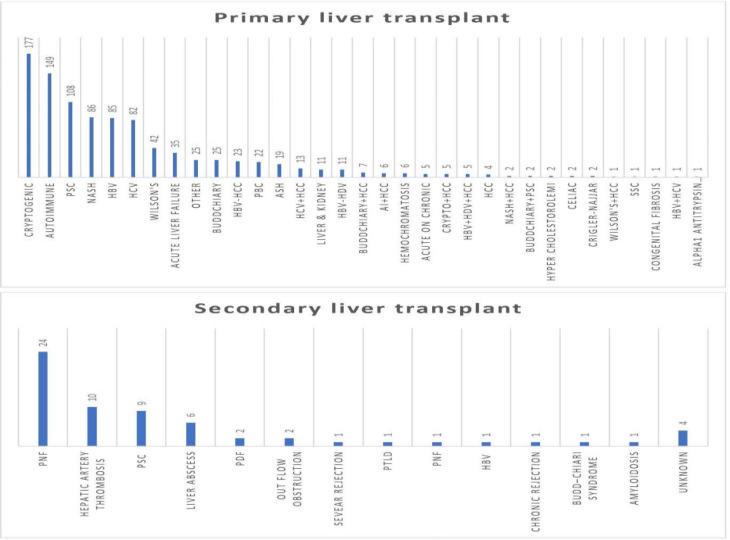
*Causes of primary liver transplant and* *re-transplant*

The most common causes of primary liver transplantation were cryptogenic cirrhosis (177; 18.32%), autoimmune (149; 15.42%), PSC (108; 11.18%), NASH (86; 8.9%), HBV (85; 8.79%), and HCV (82; 8.48%). Primary nonfunctional graft (PNF) (37.5%), hepatic arterial thrombosis (10; 15.62%), and PSC (14.06%) were the most common causes of Re-LT (figure 1).

**Figure 2:**
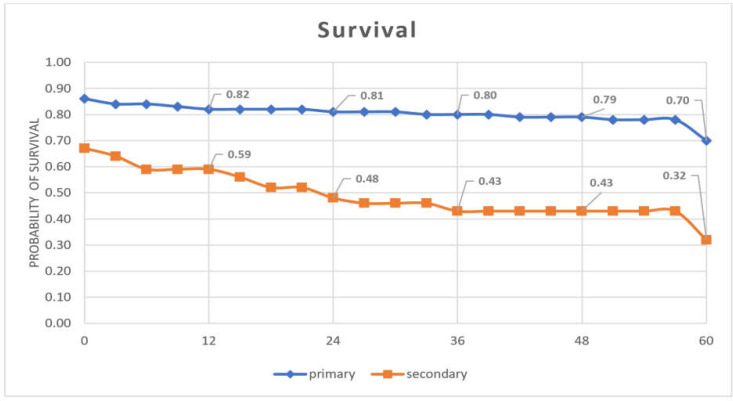
*Survival of primary liver transplant and* *re-transplant* *patient*

The survival rate of primary and Re-LT patients is demonstrated in Figure 2. The survival rate of the patients with first transplant in the first, third, and fifth years was 82%, 80%, and 70%, respectively. Survival rates in Re-LT patients in the first, third, and fifth years were 59%, 43%, and 32%, respectively. Survival in patients with Re-LT was significantly lower than in patients with primary transplantation.

Predicting variables affecting survival rates in transplant patients, Hb and ALKP variables were able to predict survival rates in Re-LT patients. From all variables monitored in the study, Hb and ALKP were able to predict survival rates and could be utilized as prognostic markers. Using the Hb marker alone and in combination with ALKP, the survival rate was predicted in 46.1% and 71% of cases, respectively.

**Table 2:** Univariant analysis for predicting factors

	Variable	Adjusted R^2^	B	β	P
Survival	Hb	0.461	366.305	0.718	0.013
	Hb ALKP	0.710	544.524 0.316	1.067 0.612	0.001 0.018

## Discussion

The current study examined the causes of Re-LT, the survival of patients with Re-LT, and the factors influencing their prognosis. The results of a study of 1,030 LT showed that the mortality rate in Re-LT patients was 54.86%, which is significantly higher than in patients with primary transplantation. 

Re-LT is performed when the previous transplant has been lost. This procedure is the only treatment for irreversible liver failure and accounts for 10-20% of LT operations worldwide (13). However, Re-LT accounted for 5.75% of all LT operations performed in the current study. This difference could be due to different criteria in the two studies for surgery, different results in the outcomes of the surgical teams, or different mortality rates in the waiting list for LT.

Primary nonfunction liver transplant (PNF) and thrombosis of the hepatic arteries are the most prevalent reasons for Re-LT in previous studies .(14)PNF, which is the most common indication of Re-LT according to the current study, is a process that begins immediately after transplantation and causes 7.3-46.3% of Re-LT indications (15). Although the cause of this phenomenon is not clear, it seems that changes in liver microcirculation cause this condition (16). Liver thrombosis in this study was the cause of 15% of Re-LT cases. These results were in line with previous studies, in which liver thrombosis accounted for 8.7% to 29.3% of Re-LT (17) .

Age and sex between the two groups of re-transplantation and primary transplantation were similar in the current study. Previous studies have shown that age is a risk factor for premature mortality (18), and Lindares et al. showed that both comorbid situations and older age concurrent with Re-LT procedure could increase the mortality rates in these patients (19). Because of the similar age and sex ratio between the groups in this study and the higher mortality rate in patients with Re-LT, it is discernible that underlying factors other than age and gender can affect the mortality rates in Re-LT patients.

Watt et al. reported lower survival for patients with recurrent MELD above 25. In the current study, patients had a MELD of over 25, and mortality was significantly higher in patients with Re-LT (20). In this study, unlike previous studies, age and bilirubin levels were not suitable variables for predicting survival in transplant patients .(21) These results are likely due to the lower number of patients with re-transplantation. Previous studies have shown that the rate of blood variables played a role in the mortality of re-transplant patients .(22) In the current study, Hb was an indicative factor in predicting survival in transplant patients.

According to the current results, although the one-year survival rate of Re-LT patients was 59%, the survival rate decreases to 32% within 5 years post-operation. The chances of survival in this study were higher than those of Rodrigo Torres-Quevedo et al. (23). In a retrospective study conducted at the University of Los Angeles, the survival rates of 1 and 5 years were reported as 62% and 47%, which is similar to the current results (24). In a prospective study, Bussutil et al. examined 450 transplant patients and reported that the survival rate of patients aged 1 to 5 years was 59% to 52%, respectively, reporting a one-year survival rate similar to the current study (2).

The results of this study showed that the survival rate in Re-LT patients is significantly lower and the mortality rate in transplant patients is significantly higher. Despite high mortality in patients with Re-LT, this treatment is still a reliable treatment in patients with liver failure.

## Conflict of interests

The authors declare that they have no conflict of interest.
